# Medical student attitudes toward video games and related new media technologies in medical education

**DOI:** 10.1186/1472-6920-10-50

**Published:** 2010-06-24

**Authors:** Frederick W Kron, Craig L Gjerde, Ananda Sen, Michael D Fetters

**Affiliations:** 1 Medical Cyberworlds, Inc., 3895 Swoboda Road, Verona, 53590, USA; 2 Department of Family Medicine, University of Wisconsin School of Medicine and Public Health, 1100 Delaplaine Ct., Madison, 53715-1896, USA; 3 Department of Family Medicine, University of Michigan, 1018 Fuller Street, Ann Arbor, 48109-1213, USA

## Abstract

**Background:**

Studies in K-12 and college students show that their learning preferences have been strongly shaped by new media technologies like video games, virtual reality environments, the Internet, and social networks. However, there is no known research on medical students' game experiences or attitudes towards new media technologies in medical education. This investigation seeks to elucidate medical student experiences and attitudes, to see whether they warrant the development of new media teaching methods in medicine.

**Methods:**

Medical students from two American universities participated. An anonymous, 30-item, cross-sectional survey addressed demographics, game play experience and attitudes on using new media technologies in medical education. Statistical analysis identified: 1) demographic characteristics; 2) differences between the two universities; 3) how video game play differs across gender, age, degree program and familiarity with computers; and 4) characteristics of students who play most frequently.

**Results:**

217 medical students participated. About half were female (53%). Respondents liked the idea of using technology to enhance healthcare education (98%), felt that education should make better use of new media technologies (96%), and believed that video games can have educational value (80%). A majority (77%) would use a multiplayer online healthcare simulation on their own time, provided that it helped them to accomplish an important goal. Men and women agreed that they were most inclined to use multiplayer simulations if they were fun (97%), and if they helped to develop skill in patient interactions (90%). However, there was significant gender dissonance over types of favorite games, the educational value of video games, and the desire to participate in games that realistically replicated the experience of clinical practice.

**Conclusions:**

Overall, medical student respondents, including many who do not play video games, held highly favorable views about the use of video games and related new media technology in medical education. Significant gender differences in game play experience and attitudes may represent male video game design bias that stresses male cognitive aptitudes; medical educators hoping to create serious games that will appeal to both men and women must avoid this.

## Background

In 2000, Charles P. Friedman published a remarkably farsighted paper that suggested that medical education had become "stuck" in space, time, and content[1]. A sweeping set of cultural and technological changes was creating digitally-savvy learners "who have experienced increasingly sophisticated video games and have spent hours with such excellent simulations as Sim City and Flight Simulator." Friedman posited that learners would immediately and intuitively appreciate that new media could enhance their medical education, and that seeing highly sophisticated medical programs lacking a correspondingly high level of technologic sophistication would likely baffle them.

"New media" refers to the many forms of electronic communication made possible by computer technology, including recreational, informational, social, cultural, pedagogical, and commercial applications that are manipulable and networkable. Examples of new media include computer and video games (both casual and serious types), virtual reality environments, social networks, web sites, mobile devices, blogs, and podcasts[2-4].

### Nine Years Later: Serious Games

Since Friedman's publication, interest in computer games as vehicles for serious scholarship has skyrocketed[5-7]. Yee successfully challenged the stereotype that online games are the exclusive purview of adolescent loners, and revealed instead that college students, CEOs, college professors, middle-aged homemakers, and retirees all belong to the gameplaying demographic[7]. Holtziger, Kickmaier-Rust and Albert demonstrated the superiority of dynamic media over static textbook lessons (passive media) proportionate to the complexity of the learning materials[8]. Gee analyzed task demands and designs of several contemporary computer games belonging to different genres and concluded that successful games incorporated learning principles that were congruent with the most broadly-supported theories of learning and cognition[9]. Schaffer elucidated how games help learners reframe their identities and interests in relation to professional communities of practice. Games that have objectives beyond entertainment--"serious" games--can provide the sort of deep, epistemic learning that traditional teaching techniques may lack[10].

Researchers at the Massachusetts Institute of Technology (MIT) and Stanford University have even suggested that implementing organizational and management standards found in massively multiplayer online role-playing games (MMOGs or MMOs) could improve leadership capabilities in real-world companies[11]. An MMOG enables hundreds or thousands of players to simultaneously interact in a persistent, virtual game world via the Internet. Indeed, IBM currently uses serious games to teach business process management, and Cisco uses serious games to teach networking concepts[12,13]. Raybourn, an expert in human-computer interaction and social-process simulations, uses virtual worlds to study intercultural communication and to mediate conflict resolution[14,15]. Economist Castronova sees MMOGs not only as leadership simulators, but also as perfect, clean laboratories for economics simulation and study[16,17].

### Millennial Medical Students

Current medical students belong to a generational cohort called "Millennials" or the "Net Generation."[18-22]. Due in part to their high degree of technological literacy, they are a radically different audience than the students of fifteen or twenty years ago. A recent National School Boards Association survey revealed that 96% of the student respondents with online access used social networking technologies such as chatting, text messaging, blogging, and visiting online communities such as Facebook and MySpace; 71% used social networking tools weekly[23]. Millennials enjoy learning about new technology through discovery, by experiencing and experimenting with it. They read less, and are more comfortable in image-rich environments than with text[24]. Their clear preference is for active, first-person, experiential learning and a level of interactivity that is absent in traditional lectures, but vibrantly present in new media technologies[25]. Thus, the growing movement towards using new media and serious games in education fits well with Millennial medical students' learning styles.

### Serious Grants

If the amount of grant-supported research is the standard by which to judge how seriously national experts view new ideas, then serious games are valued very highly by such prestigious organizations as The Robert Wood Johnson Foundation and the MacArthur Foundation."[26,27]. The International Center on Nonviolent Conflict recently funded a serious game version of the documentary *A Force More Powerful *to teach using nonviolent tactics to overcome oppression, and the European Union (EU) Framework VI Program is actively funding serious games that promote personal and social education for children. Considering the growth and success of these efforts, digital curriculum will likely evolve from an adjunct into a mainstream teaching method.

### Study Purpose

Academic leadership has called for innovative methods to enhance how medical students access the concepts that they need to become doctors[28]. New media technologies developed by the video game industry hold great promise of helping educators to meet this critical mandate. Nonetheless, the authors found no identifiable research on medical students' experiences and views about the role of new media in medical education.

The purpose of this investigation, therefore, was to examine medical students' experience with, and attitudes toward, game play. Such data can inform whether their attitudes and experiences warrant the development of new media teaching methods in medicine and, if true, to better understand how those applications should be designed so as to resonate strongly with this group of learners.

## Methods

### Design

The study design employed an anonymous, cross-sectional, web-based survey using Survey Monkey--a commercial survey delivery service[29].

### Setting and Population

Research was conducted at the University of Wisconsin-Madison (UW) and the University of Michigan (UM). The UW exempted the study from IRB review, while the UM IRB approved the study. Medical students, including graduating seniors, enrolled in these two institutions were the target population. Data collection covered April and May, 2007.

### Survey Instrument

Designed by the investigators, the survey instrument contained 30 items that included: (1) questions on demographics and four major domains, namely game-play experience and attitudes about game-play; (2) attitudes about the use of new media technology; (3) beliefs about multiplayer online simulations for healthcare education; and (4) perceived importance of acquiring specific skills, knowledge and behaviors during medical school. Most of the items were created upon consultation with experts in the area and were designed to extract general characteristics of the game-playing subpopulation of medical professionals. The UW investigator (FWK) hosted a brown-bag luncheon to discuss student interest in new health media, to raise interest in the research and to help develop survey questions. The online survey included skip patterns for items not relevant to the participant based on individual responses. For example, participants who replied that they "did not play games" were not asked to answer which games they play. Scales measuring agreement with attitudinal items were written using the four-point Likert scale format (strongly agree, agree, disagree, and strongly disagree).

### Data Collection Procedures

Data collection involved an email solicitation containing a clickable link to the Survey Monkey survey. The study investigator at each institution sent the solicitation to medical students at his institution. A reminder email was sent one and two weeks later.

### Data Analysis

The survey data were downloaded from Survey Monkey into SPSS (Statistical Package for the Social Sciences) statistical software. Frequencies and summary statistics were calculated for all variables and results presented using valid percentages. Data were organized to address the major domains of inquiry. To simplify presentation, strongly agree or agree responses and disagree or strongly disagree responses, respectively, were combined for some items.

Descriptive statistics were calculated on demographic information, age, gender, program (e.g., MD vs. MD/PhD), familiarity with level of computers, time spent playing, age started playing games, and number of games owned. Two-sample t-test or two-group chi square test were performed to identify any difference across institutions. We conducted a binary logistic regression to identify who plays video games and how video game play differs across gender, age, degree program and familiarity with computers. For the subset of people who played video games, we ran an ordinal logistic regression to identify the characteristics of those who play more frequently. To examine change in hours of game play, we collapsed "increased a lot" and "increased," and "decreased somewhat" and "decreased a lot," into the two categories called "increased" and "decreased," respectively.

We used chi-square tests to compare two independent proportions for dichotomous outcomes and two-sample t-tests for comparing two means, in order to compare various characteristics between males and females. For proportion comparisons, Fisher's exact test was used when observed proportion fell below 10% in any cell. Binary logistic regression was used to model the dichotomous outcomes with gender, institutional affiliation, familiarity level (basic/intermediate or advanced), and age as independent variables. Ordinal outcomes such as frequency of play change in playing hours and level of interest in multiplayer online healthcare simulations were analyzed using ordinal logistic regression.

## Results

### Demographics

Overall, 227 medical students responded though 10 incomplete surveys were eliminated. This report is based on data from 217 respondents, and from a subgroup of 109 respondents who were self-identified game players and provided game play data. Table 1 provides the overall demographics of the respondents. The UW provided 125 respondents and the UM provided 92. The distribution was nearly equal by gender. Approximately half the respondents (55%) were in their first two years of study. Nearly 97% of the respondents considered themselves to be intermediate or advanced users of computers. Virtually all (99.5%) reported having broadband access to the Internet. Respondents spent a mean time of 98 minutes (standard deviation, 68 minutes) in a session of video game play; the median time was 90 minutes. These numbers suggest a slight positive skew due to a few heavy gamers.

**Table 1:** Participant Demographics

(N = 217)	Total n(%)	Male n(%)	Female n(%)	P-value
School				
UM	92(42)	51(50)	41(36)	0.03
UW	125(58)	51(50)	74(64)	NS**

Familiarity with computers				
Basic & Intermediate	169(78)	62(61)	107(93)	<0.001
Advance	48(22)	40(39)	8(7)	NS

Game platforms owned*				
PDA	138(66)	68(67)	70(61)	NS
Sony PS2/PS3	32(15)	21(21)	12(10)	0.04
Microsoft X Box	17(8)	16(16)	2(2)	<0.001
Hybrid Device(e.g., PDA/phone)	11(5)	9(9)	3(3)	NS
Handheld Game	10(5)	8(8)	3(3)	NS

	Mean(SD)	N Mean(SD)	N Mean(SD)	

Age	25(3.8)	100 26(3.1)	114 25(3.6)	NS

Age started video game play	9.4(4.1)	67 8.6(3.4)	41 10.5(4.9)	0.03

Average time in one session of video gameplay(in minutes)	98(68)	67 107(70)	38 82(62)	NS

*Multiple responses were permitted; percentages do not add to 100%

**NS = Non-significant

In assessing for gender differences, we found that there was a higher proportion of males with advanced familiarity with computers (p < 0.001, Fisher's exact) and that a significantly higher proportion of males owned Microsoft's X-box. Female respondents started playing video games at a later age than male respondents (p = 0.03). There were more females from UW than from UM (p = 0.03), and more female MD/PhD respondents than male MD/PhDs (70% vs. 52%) (p = 0.008).

### Experience with Video Games

Table 2 illustrates the participants' experiences with video games. Of eight game genres presented, the three most popular were puzzle (27%), strategy (24%), and role-play (18%). Most students (70%) reported that their time spent playing games had decreased since entering professional school, though 20% reported it stayed the same, and 10% reported it had increased somewhat. The most frequently chosen reasons for playing video games were: "help me relax" (77%); "fun way to spend time with existing friends" (67%); "allow me to avoid studying" (63%); and "challenge me in problem solving" (62%).

**Table 2:** Experience with Game Play among Medical Students

N = 217	Total n(%)	Males n(%)	Females n(%)	P value
Don't play	102(47)	29(28)	72(63)	
Play game	116(53)	73(63)	43(37)	<0.001

What are your favorite types of video games?*(N = 115)				
Puzzle games	58(27)	28(38)	31(72)	0.001
Strategy games	52(24)	46(63)	7(16)	<0.001
Role-playing games	40(18)	33(45)	8(19)	<0.001
Arcade games	29(13)	18(25)	11(26)	NS**
First person shooter games	43(20)	41(56)	2(5)	<0.001
Sports games	40(18)	32(44)	8(19)	0.005
Simulation games	26(12)	16(22)	11(26)	NS
Adventure games	23(11)	14(19)	10(23)	NS
Other	12(6)	5(7)	8(19)	NS
				
How frequently do you play video games?(N = 109)				
Daily or almost daily	3(2.8)	2(3)	1(2)	NS
Several times per week	21(19.3)	17(25)	4(10)	NS
Several times a month	26(23.9)	17(25)	9(22)	NS
Rarely	54(54.1)	32(47)	27(66)	NS
How has the number of hours you spend playing video games changed since you started professional school?(N = 109)				
Increased	11(10)	4(6)	7(17)	NS
Stayed the same	22(20)	10(15)	12(29)	NS
Decreased	76(70)	54(79)	22(54)	0.01
				
I enjoy video games because*(N = 109)				
They help me to relax	84(77)	54(79)	30(73)	NS
They provide a fun way to spend time with existing friends	73(67)	51(75)	22(54)	0.007
They allow me to avoid studying	69(63)	44(65)	25(61)	NS
They challenge me (e.g., in problem solving)	67(62)	46(68)	21(51)	NS
I like exploring new worlds	59(54)	43(63)	16(39)	NS
I like the competitive aspects	56(51)	40(59)	16(39)	0.03
They make me feel less lonely when friends are not available	28(26)	18(27)	10(24)	NS
They help to release hostility	23(21)	16(23)	7(17)	NS
I learn a lot from playing video games	21(19)	19(28)	2(5)	NS
Video games are a great way to make new friends	14(13)	12(18)	2(5)	NS

*Multiple responses were permitted, percentages do not add to 100%

**NS = Non-significant

The following gender differences were also elucidated. Males are about 4.4 times more likely than females to play video games (95% CI: (2.3, 8.4)) (p <0.001). As regards favorite types of games: females are about 5.2 times more likely than males to play puzzle games (95% CI: (2, 13)) (p = 0.001); females are about 29% as likely as males to play role-playing games (95% CI: (.11, .78)) (p < 0.001); females are about 23% as likely as males to play sports games (95% CI: (.08, .64)) (p = 0.005); females are about 15% as likely as males to play strategy games (95% CI: (.05, .41)) (p < 0.001); and females are about 3% as likely as males to play shooter games (95% CI: (.005, .13)) (p < 0.001).

As to the reasons why respondents enjoy playing video games, females were: about 24% as likely as males to enjoy video games for fun (95% CI: (.09, .68)) (p = 0.007); about 35% as likely as males to enjoy the competitive aspects of the video games (95% CI: (.14, .91)) (p = 0.03); and about 13% as likely as males to enjoy the learning aspects of the video games (95% CI: (.03, .66)) (p = 0.01). Male students are more likely than females to have decreased the number of hours spent in playing video games (p = 0.01). With respect to frequency of game play, younger students and students with an advanced level of familiarity are more likely to play more frequently (p = 0.04, and 0.03, respectively).

### Video Games and Medical Education

Regardless of whether they identified themselves as game players, the respondents were very positive about the potential roles of new media technology and video games in medical education. Table 3 shows that an overwhelming majority of students liked the idea of using technology to enhance healthcare education (98%) and thought that medical education should make better use of new media technology (95%). A solid majority thought that real life is migrating online in many aspects (88%) and that video games can have educational value (80%). Thirty percent said they would like to be part of the design team that creates multiplayer online healthcare simulations. Interestingly, females are only about 39% as likely as males to believe in the potential educational value of video games (95% CI: (.17, .89)) (p = 0.03). Females are about 31% as likely as males to want to be part of a team to design an educational video game (95% CI: (.15, .64)) (p = 0.002). Also, students with a basic or intermediate level familiarity with computers are only about 24% as likely as those with an advanced familiarity to like to be part of a design team (95% CI: (.11, .54)) (p < 0.001).

**Table 3:** Medical Student Attitudes about Video Games

	Total n (%)	Agree M n (%)	F n (%)	Total n (%)	Disagree M n (%)	F n (%)	P-value
I like the idea of using technology to enhance the current healthcare education experience (N = 200)*	195 (98)	92 (99)	103 (96)	5 (3)	1 (1)	4 (4)	NS**

I think that education should make better use of new media technologies(N = 208)*	198 (96)	95 (100)	103 (99)	10 (5)	0 (0)	0 (0)	NS

I think real life is migrating online for many millions of people, in its personal, social, economic, educational and even political aspects(N = 208)	183 (88)	89 (91)	94 (86)	25 (12)	9 (9)	16 (15)	NS

I feel that video games can have educational value(N = 208)*	167 (80)	87 (89)	80 (73)	41 (19)	11 (27)	30 (11)	0.03

If multiplayer online healthcare simulation helped to accomplish a goal that was important to me, I would use it even if I had to do that with my own time(N = 200)*	154 (77)	77 (83)	77 (72)	46 (24)	16 (17)	30 (28)	NS

I would like to be part of the design team that creates a multiplayer online healthcare simulation(N = 200)	60 (30)	42 (45)	18 (17)	140 (70)	51 (55)	89 (83)	0.002

M = Males, F = Females

*() Percentages do not add to 100% due to rounding.

**NS = Non-significant

### Multiplayer Online Healthcare Simulations

Students supported using multiplayer online healthcare simulations. For example, a majority of students (77%) indicated that if a multiplayer online healthcare simulation helped to accomplish a personal goal, they would be willing to use it, even on their own time (Table 3). Eighty-five percent were either very interested or somewhat interested in multiplayer online healthcare simulations (Table 4). Females are much less interested than males (p < 0.001), and students with an advanced level familiarity are much more interested (p = 0.004) than their counterparts in multiplayer online healthcare simulations that realistically replicate the experience of what it's like to be in professional practice. The factors that would make a multiplayer online healthcare simulation most interesting included the simulation being "fun"; "helpful for developing skills and comfort in patient interactions"; "helpful for modeling the economics of different healthcare systems"; and "helpful for experiencing firsthand authentic experiences that shaped the views and values of professors they admire." There is no difference between male and female students with respect to these items. However, students with advanced level familiarity with computers are more likely than their counterparts to find multiplayer online healthcare simulations interesting if (a) it is for credit (p = 0.005), (b) it is based on authentic experience (p = 0.04), (c) it is visually crafted (p = 0.045), (d) it overcomes challenges (p = 0.02), and (e) it models politics (p = 0.05). No other statistically significant gender differences were elucidated.

**Table 4:** Beliefs about Multiplayer Online Healthcare Simulations and Relative Value of Clinical Facts, Critical Thinking and Interpersonal Skills

	Total n(%)	Males n(%)	Females n(%)	P-value
How interested are you in multiplayer online healthcare simulations that realistically replicate the experience of what it's like to be in professional practice*(N = 200)				
Very interested	55(28)	39(42)	16(15)	<0.001
Somewhat interested	116(58)	49(53)	67(63)	NS**
Not at all interested	29(15)	5(5)	24(22)	NS

Multiplayer online healthcare simulations would be interesting to me if:(N = 200)				
It was fun	193(97)	90(97)	103(96)	NS
It helped me to develop skill and comfort in patient interactions	180(90)	79(85)	101(94)	NS
It modeled the economics of different healthcare systems	166(83)	79(85)	87(81)	NS
It let me experience firsthand the authentic experiences that shaped the views and values of professors I admire				
As a course, it included in-person mentoring sessions with professors	165(83)	79(85)	86(80)	0.04
It was visually crafted and strategically designed to equal the best of entertainment properties	158(79)	77(83)	81(76)	NS
It modeled politics(e.g., letting people set health policy) in a realistic way	149(75)	75(81)	74(69)	0.045
It allowed me to meet interesting people in other health-related fields	148(74)	72(77)	76(71)	0.05
It was part of for-credit course	142(71)	72(77)	70(65)	
It let me overcome challenges in a group with my friends	134(67)	68(73)	66(62)	0.005
	133(67)	70(75)	63(59)	0.02

*Percentages do not add to 100% due to rounding

**NS = Non-significant

## Discussion

These data provide insight into medical student attitudes towards various instructional styles and methods, and towards the role that serious games and related new media technologies could play in enhancing medical education. They also reveal some important differences with regard to gender and characteristics of video games.

### Game Genres

While students reported a variety of favorite game genres, the most popular were puzzle, strategy, and role-playing games. This result suggests that students may have an affinity for cognitively challenging games. Role-playing games may have special educational utility to help students envision what their life would be like in different types of professional practice. Allowing students to step into the shoes of practitioners in different specialties, healthcare settings and economic systems, in an immersive and authentic way, could help to prospectively inform their decisions regarding which career choices would be the best fit with their values and personal characteristics[30,31].

It is important to recognize that favorite game genres can also be blended together in the creation of serious games for medical education. Very challenging abstract concepts--for example, how the nephron works or how a patient-centered medical professional works--can be made amenable to reification through hybrid systems drawn from any number of game-related new media technologies[32].

### Serious Game Design

Independent of genre, the design imperative of any serious medical game is that it should both *teach *and *be fun *. Some might question whether medical teaching can simultaneously be fun and still convey the appropriate degree of gravitas. However, consider the argument of media expert Marshal McLuhan:

"It's misleading to suppose there's any basic difference between education and entertainment. This distinction merely relieves people of the responsibility of looking into the matter. It's like setting up a distinction between didactic and lyric poetry, on the grounds that one teaches, the other pleases. However, it's always been true that whatever pleases teaches more effectively."[33].

Perhaps the most compelling testimony to "fun" as an indispensible quality of serious games comes from the failure of "Arden: The World of William Shakespeare," a multiplayer game designed to teach students about Elizabethan England while serving as a place for social-science experiments. According to the game's creator, Arden was scuttled a week after its much-heralded release principally because "It's not fun."[34-36]. That fully 97% of respondents chose "fun" as the quality that would most interest them in a multiplayer online healthcare simulation further supports this argument.

### Games, Genres and Gender

According to the Pew Internet & American Life Project, a majority of American adults age 18 and older (53%) play video games, and men (55%) are slightly more likely than women (50%) to play a digital game[37]. The fact that more males than females play video games is also supported by other research[38]. Our data shows that 53% of respondents play video games, but there is a far more pronounced male-female difference, with males being 4.4 times more likely than females to play video games. This may represent a distinct feature of the medical student demographic and should be explored further in future research.

Within the group of medical students who identified themselves as playing games, the strong female preference was for puzzle games. Other favored games also showed a male-female rift that was the greatest with respect to first person shooters and narrowed progressively for strategy games, sports games and role-playing games. In part, these data may be a function of neural sex differences between men and women[39]. For example, males are better at such tasks as mental rotation of three-dimensional objects, navigation through a route or maze, and target-directed motor skills (such as guiding or intercepting projectiles). Females are better at landmark memory (remembering details of objects seen along a route), object displacement (identifying if an object is missing or has been moved), and perceptual speed (rapidly identifying matching items based on visual cues). In puzzle games, the primary objective is figuring out a solution, which often involves solving enigmas, navigation, learning how to use different tools, and the manipulating or reconfiguring of objects[40,41]. Thus, it would appear that females gravitate away from games that are biased towards male cognitive strengths, such as first person shooter games, but that they are drawn to games that allow them to exercise control and elicit gratification through an optimal challenge based on their own cognitive strengths[42]. It is also possible that female game play predilections may stem from an aversion to games that are sexualized, or games that feature violence, which is often the case with first person shooters.

Whereas both men and women enjoy video games that challenge them, women prefer games emphasizing personal challenge, whereas men are more motivated by competition[42]. This conforms to our finding that females were about 35% as likely as males to enjoy the competitive aspects of the video games. One might expect that women self-selected for the rigors of medical training would somehow be different, perhaps more inured to competition than women at large. The fact that this does not appear to be the case may explain the high prevalence of stress and depression among women as compared with men in medical school, and the high negative impact on Health Related Quality of Life scores exhibited by female students transitioning into clinical training[43-46].

### Doctor-Patient Communication

It is heartening to note that one of the features that students would like to have situated into digital domains is skill and comfort in patient interactions. This is the foundation of ethical and professional medical practice. Fortunately, these educational needs align perfectly with current technological capabilities. MMOGs combine several new media technologies that provide sophisticated educational capabilities to help students master communication skills and professionalism through first-person experience. In "Tactical Iraqi" and "Darwars Ambush," the U.S. military employed these capabilities to train "non-kinetic engagement," helping soldiers who are deployed to hostile and culturally unfamiliar areas to become better thinkers and communicators while under stress."[47,48]. One sees here a compelling similarity to medical practice, where physicians' professional skills are regularly tested as they venture into culturally unfamiliar and high-stress, high-stakes situations. Medically themed MMOGs may have similar training utility for medical students.

### Medical Education Game Development

Our data indicate that medical students, overall, are interested in serious games and MMOs as pedagogical vehicles and specifically as epistemic constructs to help them develop ethical and professional ways of knowing, being, acting and interacting in the medical community. This information is heartening in view of both recent calls for higher quality medical education and the known difficulties that educators face in imparting ethical and professional values to medical students[49-52]. Blended learning that incorporates new media technologies with traditional approaches, e.g., didactic, small group discussions or standardized patients, can help overcome the limitations of traditional teaching environments and help meet this critical mandate.

However, the task of innovating change falls to mainstream groups of medical education policy makers who are generationally distanced from game-based and other new media technologies[53,54]. They may find fortitude by recognizing that Millennial students are very education-oriented, and that their bent towards digital media, interactivity, social environments, and hands-on, experiential learning actually offers medical educators a fantastic opportunity to create new, more powerful educational paradigms than ever before possible[55]. Our data further demonstrate that some medical students wish to participate in this creative process. Encouraging this inclination through a combination of institutional will and financial support will not only help to train today's learners, but will also cultivate tomorrow's medical educators.

### Limitations

As a non-random sample, it is possible that students who participated were self-selected individuals with an interest in gaming and new media; so the extent these data can be extrapolated to other medical students is uncertain. Since the survey was conducted near the end of the academic year, we do not know how many students received the invitation, as seniors may have already graduated, and others may have left for the summer. Thus, calculating an accurate response rate is difficult. As both host universities are research-focused, the respondents might have heightened interest in research and academics. Even considering these limitations, the results describe attitudes about new media in medical education for a substantial number of medical students.

### Future Research

Duplicating this work with medical students in other settings and countries is needed to determine if our findings generalize to a larger, more ethnically and culturally diverse medical student group. It would also be interesting to further break out game preferences according to platform (e.g., PC, console, handheld), and to correlate game attitudes and preferences with academic/clinical performance and career choice. Also useful would be to expand the inquiry to see whether games (such as MMOGs) that include social dimensions have an effect on stress and/or health-related quality of life, and if they can build social capital in medical student communities. As serious medical games (not merely simulations) are developed, it will be important to see how they correlate with current "gold standard" educational methods, and whether they are capable of creating shifts in perspective that affect how students regard and process educational experiences. Finally, further investigation into gender and game play may provide additional, important insights into women's experiences in medicine.

## Conclusions

The data obtained through this investigation supports the development of new media teaching methods in medicine; moreover, it shows how game play research can be used to better understand the medical student's mind. Like an adventure game, the research and development of serious games for medical education will offer not only interesting surprises, but also the promise of great rewards, both "in-game" and in real life. And yes, darn it ... it will be fun!

## List of Abbreviations

In this paper, we used the following abbreviations: CEO: chief executive officer; EU: European Union; K-12: kindergarten to 12^th ^grade; MMOG or MMO: Massively Multiplayer Online Role Playing Games; Net Gen: Internet Generation; UM: University of Michigan; USA: United States of America; UW: University of Wisconsin.

## Competing interests

### Financial competing interests

(FWK) This research was conducted when Dr. Kron was Family Medicine Faculty at the University of Wisconsin - Madison (UW). He subsequently founded a UW spinout corporation called Medical Cyberworlds, Inc. and currently serves as President of this entity. This company was created to explore means by which traditional medical curriculum could be enhanced and, if possible, to provide research-based means by which to enable that end. Although serious games for medical education is an area of interest, the company presently does not have any customers or products for sale, and there is no issue at present with financial gain or loss directly related to the publication of this article. The company has applied for patents, but no patentable material is included in this article. Dr. Kron has no other competing financial interests to declare. He affirms and his co-authors vouch for the fact that the conduct of this research and the resultant data and conclusions are in no way influenced by Dr. Kron's affiliation with Medical Cyberworlds, Inc.

(MDF) Dr. Fetters has served as a consultant to Medical Cyberworlds, Inc. He vouches that his role as a consultant has no influence on interpretation of the data.

(CLG) None.

(AS) None.

### Non-financial competing interests

None

## Authors' contributions

FWK, MDF and CLG participated in the conception, design of the study and creation of the web-based survey. AS, contributed to the study design. CLG and AS performed the statistical analyses. MDF and FWK drafted the manuscript. All authors participated in interpreting the data and reading, editing, and approving the final manuscript.

## Author Information

Frederick W. Kron, MD served as Assistant Professor, Department of Family Medicine, University of Wisconsin School of Medicine, at the time that this research was conducted. He subsequently founded and currently serves as President of Medical Cyberworlds, Inc.-- a company created to explore means for enhancing traditional medical curriculum with new media and to utilize research-based methods for evaluating the effectiveness of new media in medical education.

Craig L. Gjerde, PhD serves as Professor, University of Wisconsin School of Medicine and Public Health, Department of Family Medicine.

Ananda Sen, PhD serves as Research Scientist in the University of Michigan, School of Medicine, Department of Family Medicine.

Michael D. Fetters, MD, MPH, MA serves as Associate Professor, University of Michigan School of Medicine, Department of Family Medicine.

## Pre-publication history

The pre-publication history for this paper can be accessed here:

http://www.biomedcentral.com/1472-6920/10/50/prepub
